# MicroRNA-144 inhibits cell proliferation, migration and invasion in human hepatocellular carcinoma by targeting CCNB1

**DOI:** 10.1186/s12935-019-0729-x

**Published:** 2019-01-14

**Authors:** Junsheng Gu, Xiaorui Liu, Juan Li, Yuting He

**Affiliations:** Department of infectious disease, The First Affiliated Hospital of Zhengzhou University, Zhengzhou, 450052 Henan People’s Republic of China

**Keywords:** MicroRNA-144, Hepatocellular carcinoma, CCNB1, Proliferation, Migration, Invasion

## Abstract

**Background:**

Hepatocellular carcinoma (HCC) is one of the most common malignancies with a high morbidity and mortality worldwide. MicroRNAs are key regulators of HCC genesis. However, the regulatory role and underlying mechanisms of microRNA in HCC is still limited.

**Methods:**

Cyclin B1 (CCNB1) mRNA levels were examined in non-tumor and liver cancer of The Cancer Genome Atlas (TCGA) cohort. CCNB1 was knockdown to evaluate the HCC cell proliferation, migration and invasion. MicroRNA-144 targeting CCNB1 was identified with TargetScan analysis and confirmed with reporter assay. Overexpression of MicroRNA-144 was achieved using microRNA mimics and function of microRNA-144 was tested in vitro HCC cell line proliferation and in vivo tumor formation experiments.

**Results:**

Here, we found that the high level expression of CCNB1 was closely associated with poor prognosis in HCC patients. Knockdown of CCNB1 by RNA interference significantly inhibited cell proliferation, migration and invasion in HCC. Furthermore, we found that miR-144 directly targeted CCNB1 and inhibited CCNB1 expression. Moreover, in vivo experiments of subcutaneous tumor formation further demonstrated that miR-144 delayed tumor formation by negative regulation of CCNB1.

**Conclusion:**

Therefore, we conclude that microRNA-144/CCNB1 axis plays an important role in human HCC. Therapies targeting microRNA-144 could potentially improve HCC treatment.

**Electronic supplementary material:**

The online version of this article (10.1186/s12935-019-0729-x) contains supplementary material, which is available to authorized users.

## Background

Hepatocellular carcinoma (HCC) is one of the most common malignancies with a high morbidity and mortality worldwide [1, 2]. However, the precision pathogenesis of HCC is unclear and the effective prevention and treatment are still lacking. Thus, studying the molecular mechanism of HCC proliferation and metastasis, accurately predicting prognosis for patients, preventing HCC metastasis, and taking effectively preventive measures could be a breakthrough to improve the efficacy of prognosis and treatment.

Cyclin B1 (CCNB1), which expression varies periodically throughout the cell cycle is a key initiator and quality control step of mitosis [3]. Recent studies have shown that CCNB1 is highly expressed in several different human cancers including breast cancer, cervical cancer, lung cancer and melanoma [4–7]. Porter et al. found that the inhibition of CCNB1 nuclear export could induce apoptosis and the accumulation of CCNB1 in the nucleus was sufficient to trigger apoptosis [8]. CCNB1 has also been demonstrated to have significant predictive power in overall survival of ER^+^ breast cancer patients and HBV-related HCC recurrence [9, 10]. However, the detailed functions of CCNB1 in HCC and how CCNB1 is regulated are still remaining elusive.

MicroRNAs (miRNAs) are an endogenous small non-coding RNAs, which regulates a broad-spectrum of genes involved in developmental and oncogene pathways at the post-transcriptional level. MiRNA can participate in many cellular processes such as cell cycle, proliferation, apoptosis and metastasis [11]. Several studies have demonstrated that miRNAs play important roles in hepatocarcinogenesis and malignant transformation. MicroRNA-144 (miR-144) is a highly conservative miRNA that participates in the process of tumor genesis and development in different cancers [12, 13]. It was reported that miR-144 suppressed cell proliferation, migration, and invasion in hepatocellular carcinoma by targeting SMAD4 [14].

In this study, we firstly analyzed the Cancer Genome Atlas (TCGA) database and found that the expression of CCNB1 was abnormally elevated and closely related to the malignant phenotype. We confirmed that CCNB1 knockdown significantly increased HCC cell apoptosis, while cell migration and invasion was decreased. Furthermore, we found that miR-144 directly inhibited CCNB1 expression by binding to CCNB1 3′-UTR. Consistently, the expression of miR-144 significantly decreased in liver cancer tissue compared with that in non-tumor tissue. Overexpression of miR-144 in HCC cell lines inhibited the cell proliferation in vitro and delayed the subcutaneous tumor formation in vivo. In conclusion, we demonstrated that miR-144, serving as tumor suppressor, played an inhibit role in the occurrence and development of HCC by negative regulation of CCNB1 expression, thereby contributing to the progress of HCC. MiR-144/CCNB1 pathway plays an important role in the development of HCC, which provides a potential promising target for HCC treatment.

## Materials and methods

### TCGA database analysis

The liver cancer gene expression profiles in the TCGA database were downloaded using the R package TCGA-assembler, including 424 samples of mRNA expression profiles (374 cases of liver tumor specimens, 50 cases of adjacent tumor specimens). Expression profiles and gene annotation files were imported by BRB-Arraytools (version V4.5.0 Beta 2) to get the gene expression value. Follow-up data were also downloaded for matching and subsequent analysis.

### Cell culture

HEK293T cells, Human HCC cell line, HepG2 and 7221 were from the Institute of Biochemistry and Cell Biology, Chinese Academy of Sciences (Shanghai, China). The cells were maintained in DMEM medium (Thermo Fisher, Waltham, MA, USA), supplemented with 10% fetal bovine serum (FBS; Hyclone, USA), 100 U/mL penicillin and 100 μg/mL streptomycin.

### Mice and tumor model

All animals were treated in accordance with the Guide for the Care and Use of Laboratory Animals, and all experiments were approved and carried out according to the guidelines of the Ethics Committee of Zhengzhou University. Male BABL/c nude mice (5–6 week old, 18–22 g weight), maintained under a specific pathogen-free condition. SMMC-7721 cells were stably infected with/without the miR-144 overexpression mimic vectors. A total of 5 × 10^6^ viable cells were injected into the right flanks of nude mice. Tumor sizes were measured using a vernier caliper every 5 days, and tumor volume was calculated using the formula: volume = 1/2 × length × width^2^. At 30 days after implantation, the mice were killed, tumors dissected, and tumor weights were measured.

### siRNA/miRNA transfection

siRNA targeting CCNB1 (CCNB1-siRNA, GAAUUCUGCACUAGUUCAA), Scramble siRNA control (Scramble-siRNA, GAUUAGUCACCUGACUAUA), miR-144 mimics (GGAUAUCAUCAUAUACUGUAAG), miR-144 inhibitor (CUUACAGUAUAUGAUGAUAUCC) and Scramble controls (GAGUACUACUUAUACAGUAUAG) were designed and synthesized by Shanghai GenePharma Company (Shanghai, China). Cells were seeded into 96-well plates in antibiotic-free growth medium at the density of 4 × 10^3^ cells per well. Transfection with was performed when the cells reached 70–80% confluence using Lipofectamine 2000 (Invitrogen, USA) in accordance to the manufacturer’s protocol.

### Real-time quantitative PCR

Total RNA extraction was extracted from the cell lines using Trizol reagent (Invitrogen, USA). Complementary DNA was synthesized using AMV reverse transcriptase (GIBCO, USA) through reverse transcription. PCR reactions were performed using following primers: 5′-GACCTGTGTCAGGCTTTCTCTG-3′ (forward) and 5′-GGTATTTTGGTCTGACTGCTTGC-3′ (reverse) for CCNB1; 5′-CAGGAGGCATTGCTGATGAT-3′ (forward) and 5′-GAAGGCTGGGGCTCATTT-3′ (reverse) for GAPDH. Relative expression levels of CCNB1 were normalized to endogenous control GAPDH using 2^−ΔΔCT^ methods. miRNA were extracted using an miRNA isolation kit miRNeasy kit (Qiagen, Germany) according to the manufacturer’s protocol.

### Cell proliferation assay

Cell proliferation was determined using the MTT assay. Cells were seeded at a density of 3 × 10^3^ per well in 96-well plates, and each well was transfected with CCNB1-siRNA, miR-144 mimics, miR-144 inhibitor or the proper negative control. At 48 h after transfection, 3-(4,5-dimethylthiazol-2-yl)-2,5-diphenyl-tetrazolium bromide (MTT) (Sigma-Aldrich, USA) solution was used to replace culture medium and incubated for 4 h. A microplate reader (Bio-Rad, Hercules, CA) measured the absorbance at 570 nm for all the samples.

### Apoptosis analysis by Annexin-V FITC/PI double staining

The HCC cell line HepG2 and SMMC-7721 were transfected with CCNB1-siRNA or negative control (Scramble-siRNA). To identify apoptotic cells, Annexin V and PI staining was performed using an Annexin V-FITC Apoptosis Detection kit (Becton, Dickinson and Company, San Jose, CA, USA) after 48 h. Apoptosis was detected using a Calibur Flow Cytometer (Becton). Apoptotic cells were defined by positive staining of Annexin V and negative staining of PI.

### Cell invasion assay

The invasion assay was performed using a transwell chamber, consisting of 8 mm membrane filter inserts (Corning Incorporated, Corning, NY, USA) coated with Matrigel (BD Biosciences, San Jose, CA, USA). Briefly, cells were trypsinized and suspended in serum-free medium. Next, 2 × 10^4^ cells were added to the upper chamber, and the lower chamber was filled with medium containing 10% fetal bovine serum. After 36 h of incubation, cells that had invaded the lower chamber were fixed with 4% paraformaldehyde, stained with hematoxylin, and counted using a microscope.

### Wound-healing assay

Wound-healing assay was performed using HepG2 and SMMC-7721 cells. Cells were trypsinized and seeded in equal numbers into six-well tissue culture plates, and allowed to grow until confluent (approximately 24 h). Following serum starvation for 24 h, an artificial homogenous wound (scratch) was created onto the cell monolayer with a sterile 100 μL tip. After scratching, the cells were washed with serum-free medium, complete media was added, and microscopic images (20× magnification) of the cells were collected at 0, 36, and 48 h.

### Luciferase reporter assay

The 3′-untranslated region (3′-UTR) of CCNB1 mRNA, which contained the predicted miR-144 binding site, was amplified by PCR using the Takara PCR Amplification Kit (Takara, Dalian, China). The following PCR primers were used for cloning 3′-UTR of CCNB1 mRNA (Forward: AAGGCTGTGGCAAAGGTGTAAC; Reverse: TCCCCAGGTAAACCAAAAGGAGT). The Quick Mutagenesis Stratagene kit (Stratagene, La Jolla, CA) was used to create mutant 3′-UTR of CCNB1 mRNA. The PCR products were then enzymatically digested and cloned into the pGL3-CCNB1-3′-UTR vector (Promega, USA). Cells (5 × 10^4^) were seeded in 24-well plates and cultured for 24 h. Reporter plasmids (200 ng the pGL3-CCNB1-3′-UTR or pGL3-CCNB1-3′-UTR-mut, 1 ng pRL-TK renilla plasmid, Promega) and 100 nmol/L miR-144 mimics were co-transfected into HEK293 cells mediated by Lipofectamine 2000 (Invitrogen). After 48 h, the cells were lysed and reporter activity was determined using a Dual Luciferase Reporter Assay Kit (Promega, Madison, WI, USA), according to the manufacturer’s instructions.

### Western blotting

Western blotting was performed using standard methods. Membranes were probed with polyclonal rabbit antibodies against anti-CCNB1 (Abcam, Cambridge, MA, USA). The proteins on membranes were then stripped and re-probed with an anti-beta-actin rabbit polyclonal antibody (Abcam) as a loading control. The blot was developed using enhanced chemiluminescence solution (Beyotime, Haimen, China) and photographed using the FluorChem imaging system (Alpha Innotech Corp., San Leandro, CA, USA). The intensity of each spot was analyzed with AlphaEaseFC software.

### Ki67 immunohistochemistry staining

To evaluate tumor tissue histological changes, tumor sections were processed and immunohistochemical staining of the paraffin sections was performed using a microwave-based antigen retrieval technique, specimen slides were incubated overnight at 4  °C with primary antibodies that were raised against Ki67 (D2H10, Cell Signaling Technology), then Dako LSAB2 System-HRP (K0675), and Dako Liquid DAB + Substrate Chromogen System (K3468).

### Statistical analysis

Unpaired two-tailed Student’s *t* test was used for calculation of *p* values between two groups. One-way analysis of variance (ANOVA) with the Tukey post hoc test and two-way analysis of variance (ANOVA) followed by a Bonferroni post hoc test were used tfor comparison between multiple groups. All *p* values < 0.05 were considered significant. Statistical analysis was performed with Graphpad Prism 6.

## Results

### Bioinformatics analysis of CCNB1 expression in HCC tissues and normal liver tissue

**Fig. 1:**
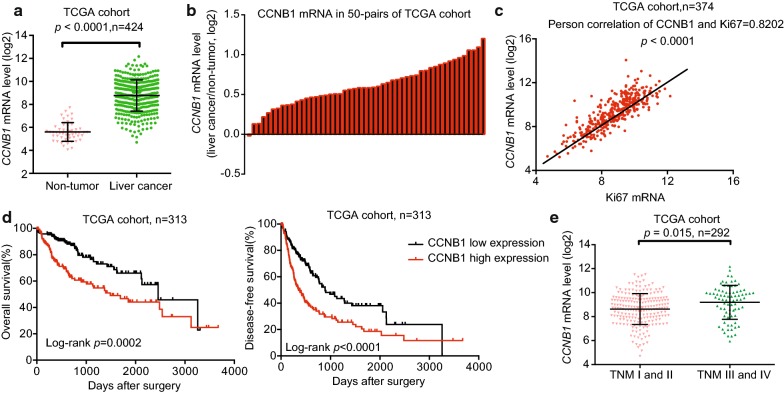
Bioinformatics analysis of CCNB1 in HCC tissues and normal liver tissues using TCGA cohort public database. **a** Comparison of CCNB1 mRNA levels in HCC tissue and non-tumor tissues in the TCGA database. The expression of CCNB1 was normalized using a logarithm. *p* < 0.0001, n = 424. **b** Comparison of CCNB1 mRNA levels in 50 pairs of HCC tissues and adjacent non-tumor tissues. **c** Pearson correlation between CCNB1 mRNA level and proliferation marker Ki67 mRNA level. *p* < 0.0001, n = 374. **d** Kaplan–Meier analysis of the overall survival and disease-free survival time of patients with high or low CCNB1 expression, n = 313. **e** The expression of CCNB1 mRNA level in HCC patients with different TNM stages (*p* = 0.015, n = 292)

CCNB1 was reported highly expressed in several different human cancers [15]. To study CCNB1 in HCC, we first analyzed mRNA expression in HCC tissues and normal liver tissues based on the liver cancer gene expression profiles in the TCGA database. Compared with the normal liver tissue, the mRNA expression levels of CCNB1 were significantly higher in HCC tissue (Fig. 1a, b). As shown in Fig. 1c, the proliferation marker Ki67 showed a pearson correlation coefficient of 0.8202 between CCNB1 mRNA level and Ki67 mRNA level, indicating that CCNB1 level was related with cell proliferation in HCC patients. Furthermore, Kaplan–Meier analysis showed that the overall survival and disease-free survival time of patients with low CCNB1 levels was significantly longer than patients with high CCNB1 levels (Fig. 1d). Interestingly, HCC patients with TNM III/IV had significantly higher CCNB1 levels compared with that of HCC patient with TNM I/II (Fig. 1e), suggesting that the expression level of CCNB1 was closely related to the prognosis of HCC patients.

### CCNB1 knockdown promotes apoptosis and suppresses proliferation in HCC cell line HepG2 and SMMC-7721

**Fig. 2:**
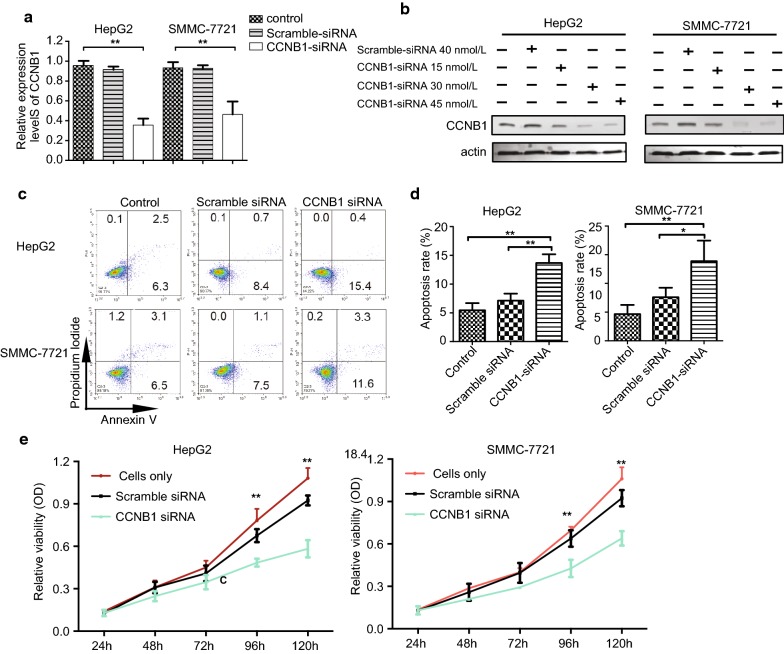
CCNB1 knockdown promotes apoptosis and suppresses proliferation in HCC cell line HepG2 and SMMC-7721. **a** Real-time PCR analysis of CCNB1 mRNA levels and **b** western blot analysis of CCNB1 protein levels in HCC cell line Hep-G2 and SMMC-7721 with no transfection (control), transfected with control siRNA (Scramble-siRNA) or CCNB1 siRNA (CCNB1-siRNA). Bargraphs in **a** represent normalized data from three independent experiments. **p < 0.01 vs. control. **b** Actin expression levels were detected as an endogenous control. Experiments were repeated twice and representative data was shown. **c**, **d** HepG2 and SMMC-7721 cells were transfected with either Scramble siRNA or CCNB1 siRNA at 50 nM. 48 h after transfection, cells were subjected to apoptosis detection using flow cytometry by Annexin-V/Propidium Iodide (PI) staining. **c** Representative dot plots show the percentages of Annexin V +/PI- apoptotic cells. **d** Bar graphs represent the mean ± SD from 3 independent experiments. *p < 0.05, **p < 0.01 vs. Control or Scramble siRNA. **e** HepG2 and SMMC-7721 cells were transfected with either Scramble siRNA or CCNB1 siRNA at 50 nM. Cell proliferation at different time points was measured by MTT assay. Bar graphs represent the mean ± SD from triplicate wells and experiments were repeated three times. **p < 0.01 vs. Cell only group

To study the function of CCNB1 in HCC, CCNB1-siRNA was transfected into hepatoma cell line HepG2 and SMMC-7721 cells. The CCNB1 knockdown efficiency was confirmed at both mRNA level and protein level (Fig. 2a, b). As shown in Fig. 2c, d, CCNB1-siRNA was transfected into HepG2 and SMMC-7721 cells, cell apoptosis was analyzed 48 h later. The apoptosis rate increased dramatically in CCNB1 knockdown group. In addition, MTT assay showed significantly lower cell proliferation in both HepG2 and SMMC-7721 cells after CCNB1 knockdown (Fig. 2e). Taken together, it is clear that CCNB1 knockdown suppresses cell proliferation and promotes apoptosis in HCC cell line HepG2 and SMMC-7721.

### CCNB1 knockdown inhibits HCC cell migration and invasion

**Fig. 3:**
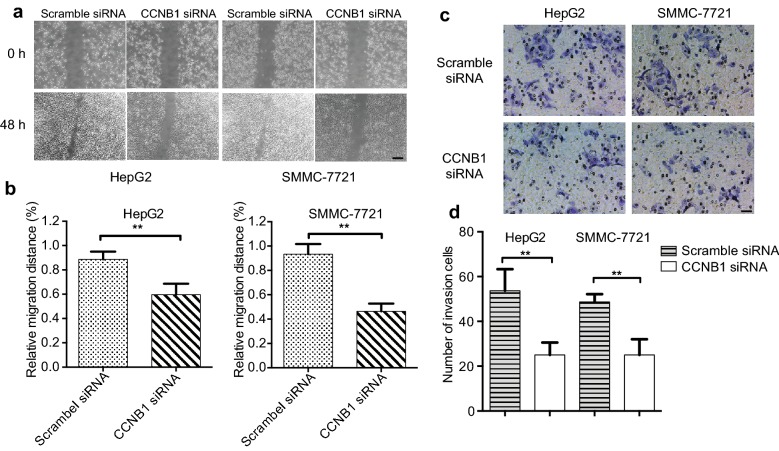
CCNB1 knockdown inhibits HCC cell migration and invasion. **a** HepG2 and SMMC-7721 cells were transfected with either Scramble-siRNA or CCNB1 siRNA at 50 nM. Micrographs of HepG2 and SMMC-7721 cells at 0 h and 48 h after wounding. **b** Migration distance of HepG2 and SMMC-7721 cells for 48 h was calculated and bar graphs represent the mean ± SD from 3 independent experiments. ^**^p < 0.01 vs. NS-siRNA. **c**, **d** Transwell Cell invasion activity was evaluated by Matri-gel invasion assay. The number of invading cells was counted in three images per membrane by microscopy using a ×20 objective, scale bar = 100 μm. Experiments were performed in triplicate and repeated at least three times. **p < 0.01 vs. NS control

To further investigate the biological significance of CCNB1 in HCC, we detected the CCNB1 on migration and invasion of HCC cells. Wound-healing assay showed that the mobility of HepG2 and SMMC-7721 cells decelerated in CCNB1-siRNA group within 48 h compared with control group (Fig. 3a, b). Transwell with Matrigel showed that CCNB1 knockdown led to a significant decrease in invasive potential of HepG2 and SMMC-7721 cells (Fig. 3c, d). In summary, CCNB1 knockdown inhibits HCC cell migration and invasion.

### MiR-144 inhibits CCNB1 expression by binding to CCNB1 3′-UTR

**Fig. 4:**
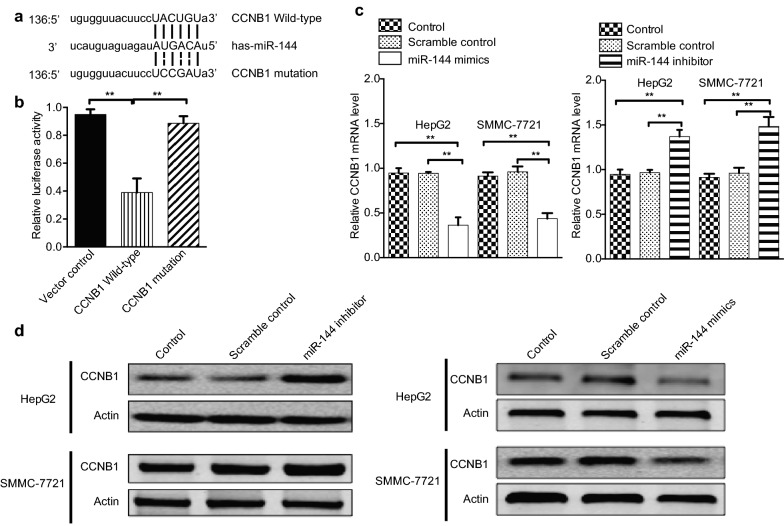
miR-144 inhibits CCNB1 expression by binding to CCNB1 3′-UTR CCNB1. **a** Diagram of the putative binding sites of miR-144 on the 3′-UTRs of CCNB1 and the mutated sequence of 3′ UTRs of CCNB1. **b** The WT 3′-UTR or mutated 3′-UTR of CCNB1 was fused to the luciferase coding region and co-transfected into HEK293T cells with miR-144 mimics. Relative luciferase activity was determined 48 h after transfection. Data are expressed as mean ± SD from 3 independent experiments. **p < 0.01 vs. Vector Control. **c** RT-PCR analysis of the effect of CCNB1 expression in HepG2 and SMMC-7721 cells after transfection with miR-144 mimics. GAPDH expression levels were detected as an endogenous control. Data are expressed as mean ± SD. Experiments were performed in triplicate and repeated at least three times. **p < 0.01 vs. control. **d** Western blot analysis of the CCNB1 expression in HepG2 and SMMC-7721 cells after transfection with miR-144 mimics or miR-144 inhibitor. Actin expression levels were detected as an endogenous control. Experiments were repeated twice and representative data was shown

Several studies have shown that miRNA regulates CCNB1 expression level, such as miR-744, miR-1186 and miR-466d-3p [16]. We integrated bioinformatics algorithms, including TargetScan, miRwalk and miRanda, to predict the potential miRNA targeting CCNB1 (Additional file 1: Figure S1). According to the prediction, CCNB1 has the putative miR-144 binding site that maps to the 3′-UTR (Fig. 4a). To further validate the prediction results, we constructed the luciferase reporters carrying the wild type and mutant type of CCNB1 3′-UTR (Fig. 4a). As shown in Fig. 4b, luciferase assays indicated that the wild type of 3′-UTR caused a significant reduction in luciferase activity, whereas mutation of the key seed region in the 3′-URT of CCNB1 showed no difference in the luciferase activity compared with the control. Real-time PCR analysis suggested that treatment with miR-144 mimics significantly down-regulated the CCNB1 mRNA level in HepG2 and SMMC-7721 (Fig. 4c). The results were further confirmed by western blot analysis. MiR-144 mimics treatment markedly inhibited CCNB1 protein level while miR-144 inhibitor enhanced CCNB1 protein level (Fig. 4d). Collectively, these results demonstrated that miR-144 might suppress the expression of CCNB1 through targeting the 3′-UTR.

### MiR-144 mimics elicit similar proliferation repressive effect in HCC cell

**Fig. 5:**
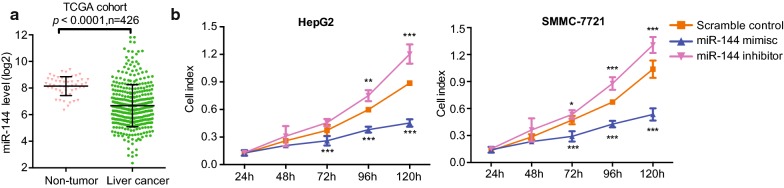
MiR-144 mimics elicit similar proliferation repressive effect in HCC cell. **a** miR-144 expression levels were analyzed in liver cancer or non-tumor tissues of TCGA cohort. **b** CCNB1-siRNA HepG2 and SMMC-7721 cells were transiently transfected with miR-144 mimics or inhibitor, and the cell proliferation was measured by MTT at different time points. Bar graphs represent the mean ± SD from triplicate wells and experiments were repeated three times. *p < 0.05, **p < 0.01, ***p < 0.001 vs. Scramble control

Since CCNB1 was a target of miR-144, we next examined miR-144 expression levels in TCGA cohort. As shown in Fig. 5a, miR-144 was significantly lower in HCC compared with that in non-tumor tissue. MiR-144 mimics transfection in HCC Cell line HepG2 and SMMC-7721 inhibited cell proliferation, which was presenting a similar effect after CCNB1 knockdown (Fig. 5b). Reversely, miR-144 inhibitor enhanced cell proliferation in HepG2 and SMMC-7721 cells (Fig. 5b).

### MiR-144 overexpression suppresses the tumor formation of SMMC-7721 cells in nude mice

**Fig. 6:**
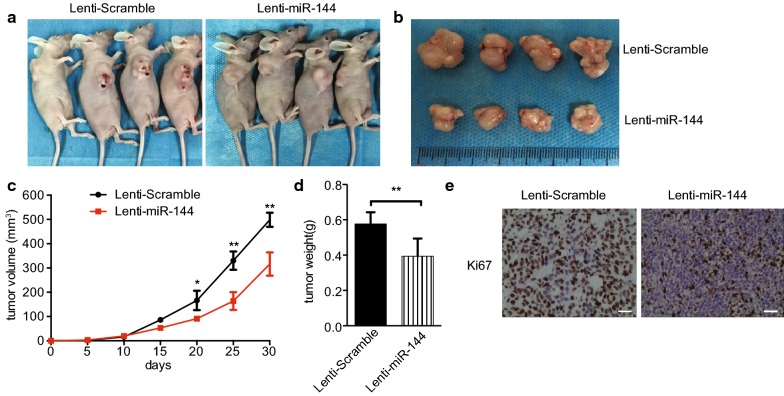
MiR-144 overexpression suppresses the tumor formation of SMMC-7721 cells in nude mice. **a**, **b** Photographs of nude mice and tumor tissues from different groups at day 30. **c** Growth curves of tumor volumes in xenografts of nude mice were determined based on tumor volume measured every 5 days for 30 days (n = 4). **d** Tumor weights of different groups at day 30 (n = 4). Data in **a**–**d** are representative of two independent experiments. *p < 0.05, **p < 0.01 vs. Lenti-Scramble, **p < 0.01 vs. Lenti-Scramble. **e** Representative immunohistochemistry images of Ki67 staining of tumor sections obtained from the Lenti-Scramble and Lenti-miR-144 mouse groups. Magnification ×200, scale bar = 10 μm

To further examine the function of miR-144 on HCC, we constructed miR-144 overexpression lentivirus vector. Relative miR-144 levels in HCC cell line SMMC-7721 were confirmed using real-time PCR (Additional file 1: Figure S2a). Consistently, overexpression of miR-144 in SMMC-7721 cells inhibited CCNB1 protein level (Additional file 1: Figure S2b). Nude mice were inoculated with HCC cell SMMC-7721 with miR-144 overexpression or scramble control cells. The survival time of nude mice and tumor growth curve were monitored and recorded. As shown in Fig. 6a–d, mice inoculated with SMMC-7721 cell overexpressing miR-144 had markedly smaller size of tumors. Immunohistochemistry results confirmed that the expression of Ki67 was decreased in tumor with miR-144 overexpression (Fig. 6e). Taken together, in vivo experiments of subcutaneous tumor formation further verified the role of miR-144-CCNB1 pathway in HCC.

## Discussions

Accumulating evidences suggested that miRNAs are important regulators in various cellular processes and are recently extensively studied in the development of HCC [17–20]. However, the precise role of miRNAs in the development of HCC is still largely unclear as a single miRNA could have multiple targets and a single mRNA could be targeted by various miRNAs [21]. Further understanding the functions of miRNAs in HCC helps to better reveal the underlying mechanism of HCC pathogenesis and progression. In this study, we demonstrated that miR-144 inhibited the proliferation, migration and invasion of HCC cells in vitro by targeting CCNB1. In vivo tumor inoculation nude mice model confirmed that overexpression of miR-144 significantly inhibited growth of HCC tumors. Thus, our results suggest that miR-144/CCNB1 axis could not only be used as a novel biomarker but also a potential gene therapeutic target of HCC.

CCNB1 regulates the G2 to M phase transition in mitosis. Overexpression of CCNB1 is correlated with poor survival in most solid tumors, which suggests that the expression status of CCNB1 is a significant prognostic parameter in solid tumors [22]. Na Chai et al. reported that Foxm1 played an important role in proliferation of HCC via transcriptional activation of CCNB1 expression, which suggested the Foxm1-CCNB1 axis could be a potential target for the treatment of HCC patients [23]. CDCA5 was found to be positively associated with increased CDK1 and CCNB1 expression in HCC tissues [24]. Interestingly, integrated bioinformatics analysis identified CCNB1 as a potential therapeutic target in HCC [25]. In this study, we explored liver cancer expression profiles from TCGA public databases and found the expression level of CCNB1 was abnormally elevated in hepatocarcinoma tissues. Kaplan–Meier analysis showed that the survival time of patients with low expression of CCNB1 was significantly better than that of patients with high expression of CCNB1, suggesting that the expression of CCNB1 is closely related to the clinical prognosis of patients with liver cancer. Meanwhile, further cell function tests confirmed that the proliferation of hepatocellular carcinoma cell lines decreased significantly after CCNB1 knockdown, and the migration and invasion ability and tumorigenic ability decreased significantly. The apoptosis rate of hepatoma cell lines was significantly increased after CCNB1 gene knockdown, suggesting CCNB1 played an important role in the occurrence and development of liver cancer.

As a mechanistic study to find the molecular mechanism of CCNB1 regulation based on its abnormally high expression in liver cancer, we also conducted bioinformatical analysis to identify the miR-144 was targeting CCNB1 by binding to its 3′ UTR. Previous studies demonstrated that miR-144 expression was decreased in various cancers, including gastric cancer, breast cancer and bladder cancer [12, 13, 26]. Our analysis revealed that the expression level of miR-144 was significantly decreased in liver cancer tissues, and the survival time of liver cancer patients with low expression of miR-144 was significantly worse than that of patients with high expression of miR-144, suggesting miR-144 may also be involved in HCC. Overexpression of miR-144 in hepatocarcinoma cells has similar effects on hepatoma cell proliferation, migration and invasion as CCNB1 knockdown, suggesting that miR-144/CCNB1 regulatory axis may play an important role in the occurrence and development of liver cancer. As the occurrence and development of tumors are complicated biological processes which are regulated by multiple factors in tumor cells and tumor microenvironment. The effect of miR-144 overexpression on the metastasis ability of hepatoma cells was also confirmed by the mouse tumor-bearing experiment.

However, miR-144-CCNB1 axis in the occurrence and metastasis of liver cancer, and further substantial follow-up studies still need further investigation. Further, it should also be noted that miR-144 regulate a broad-spectrum of biological processes and the targets of miR-144 is not limited to oncogenes. Moreover, CCNB1 is involved in important processes such as mitogenesis and the side effects of miR-144 gene therapy should also be scrutinized.

## Conclusion

In sum, we found that the high level expression of CCNB1 was closely associated with poor prognosis in HCC patients. Knockdown of CCNB1 by RNA interference significantly inhibited cell proliferation, migration and invasion in HCC. Bioinformatics study identified that miR-144 directly targeted CCNB1 and inhibited CCNB1 expression. In vivo experiments of subcutaneous tumor formation further demonstrated that miR-144 suppressed tumor formation by negative regulation of CCNB1. Therefore, these evidences suggest that microRNA-144/CCNB1 axis is an important diagnostic and therapeutic potential target in HCC treatment.

## Additional files

**Additional file 1: Figure S1.** miRNA targeting CCNB1 was screened by TargetScan, miRwalk and miRand.

**Additional file 2: Figure S2.** Overexpression of miR-144 using lentivirus vector inhibited CCNB1 expression in SMMC-7721 cells. Lentivirus vector Lenti-NC or Lenti-miR-144 was constructed and infected SMMC-7721 cells. (A) the relative miR-144 expression level was examined by RT-PCR and (B) the CCNB1 protein level was examined by western blot. Actin was used as internal control. Experiments were repeated twice and representative data was shown.
