# Supplementary material for: MicroRNA-144 inhibits cell proliferation, migration and invasion in human hepatocellular carcinoma by targeting CCNB1
Source: Cancer Cell Int. 2019 Jan 14;19:15. doi: 10.1186/s12935-019-0729-x (PMC6332595; doi:10.1186/s12935-019-0729-x)
Supplement: Supplementary file 1 — Additional file 1: Figure S1. miRNA targeting CCNB1 was screened by TargetScan, miRwalk and miRand. [file 12935_2019_729_MOESM1_ESM.pdf]

Gene Name	MicroRNA	StemLoop ID	miRanda	miRDB	miRWalk	RNA22	Targetscan	SUM
CCNB1	hsa-miR-496	hsa-mir-496	1	1	0	0	1	3
CCNB1	hsa-miR-548h	hsa-mir-548h-4	1	1	0	0	1	3
CCNB1	hsa-miR-607	hsa-mir-607	1	1	0	0	1	3
CCNB1	hsa-miR-181c	hsa-mir-181c	1	1	0	0	1	3
CCNB1	hsa-miR-181b	hsa-mir-181b-2	1	1	0	0	1	3
CCNB1	hsa-miR-548c-5p	hsa-mir-548c	1	1	0	0	1	3
CCNB1	hsa-miR-181d	hsa-mir-181d	1	1	0	0	1	3
CCNB1	hsa-miR-548i	hsa-mir-548i-4	1	1	0	0	1	3
CCNB1	hsa-miR-548j	hsa-mir-548j	1	1	0	0	1	3
CCNB1	hsa-miR-548b-5p	hsa-mir-548b	1	1	0	0	1	3
CCNB1	hsa-miR-379	hsa-mir-379	1	1	0	0	1	3
CCNB1	hsa-miR-548d-5p	hsa-mir-548d-2	1	1	0	0	1	3
CCNB1	hsa-miR-548n	hsa-mir-548n	1	1	0	0	1	3
CCNB1	hsa-miR-520g	hsa-mir-520g	1	1	0	0	1	3
CCNB1	hsa-miR-181a	hsa-mir-181a-1	1	1	0	0	1	3
CCNB1	hsa-miR-520h	hsa-mir-520h	1	1	0	0	1	3
CCNB1	hsa-miR-892b	hsa-mir-892b	1	1	0	0	1	3
CCNB1	hsa-miR-548a-5p	hsa-mir-548a-3	1	1	0	0	1	3
CCNB1	hsa-miR-559	hsa-mir-559	1	1	0	0	1	3
CCNB1	hsa-miR-144	hsa-mir-144	1	0	0	0	1	2

Additional File 1: Figure S1
