# Supplementary figures and images for: MicroRNA-144 inhibits cell proliferation, migration and invasion in human hepatocellular carcinoma by targeting CCNB1
Source: Cancer Cell Int. 2019 Jan 14;19:15. doi: 10.1186/s12935-019-0729-x (PMC6332595; doi:10.1186/s12935-019-0729-x)

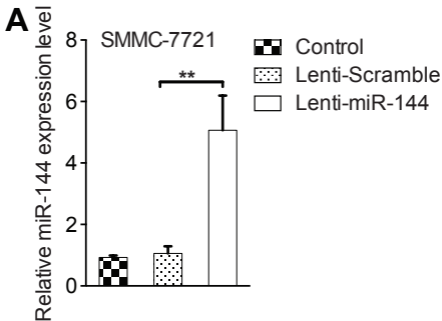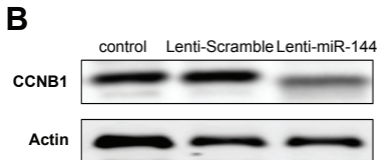

**Additional File 2: Figure S2**

Supplement: Supplementary file 2 — Additional file 2: Figure S2. Overexpression of miR-144 using lentivirus vector inhibited CCNB1 expression in SMMC-7721 cells. Lentivirus vector Lenti-NC or Lenti-miR-144 was constructed and infected SMMC-7721 cells. (A) the relative miR-144 expression level was examined by RT-PCR and (B) the CCNB1 protein level was examined by western blot. Actin was used as internal control. Experiments were repeated twice and representative data was shown. [file 12935_2019_729_MOESM2_ESM.pdf]
